# Determinants of satisfaction with cosmetic outcome in breast cancer survivors: A cross-sectional study

**DOI:** 10.1371/journal.pone.0193099

**Published:** 2018-02-21

**Authors:** Peh Joo Ho, Mikael Hartman, Danny A. Young-Afat, Sofie A. M. Gernaat, Soo Chin Lee, Helena M. Verkooijen

**Affiliations:** 1 Saw Swee Hock School of Public Health, National University of Singapore, Singapore, Singapore; 2 Department of Surgery, National University of Singapore, Singapore, Singapore; 3 Imaging Division, University Medical Center Utrecht, Utrecht, The Netherlands; 4 National University Cancer Institute, National Health System, Singapore, Singapore; University of Zurich, SWITZERLAND

## Abstract

Little research has been done into cosmetic outcomes in non-Western breast cancer populations. We aimed to study the prevalence and determinants of dissatisfaction with cosmetic outcome after breast cancer surgery of Asian breast cancer survivors, and its association with patient-reported anxiety, depression, and quality of life. In a hospital-based cross-sectional study, 384 breast cancer survivors of at least 12 months after diagnosis completed questionnaires on satisfaction with cosmetic appearance, quality of life (EORTC-QLQ-C30) and anxiety and depression (HADS). Cumulative logit models were used to examine the adjusted association between dissatisfaction with cosmetic appearance and demographic and clinical characteristics. Kruskal-Wallis test was used to test for associations between dissatisfaction with cosmetic appearance and patient-reported outcomes. Overall, 20% of women reported to be (very) dissatisfied with cosmetic appearance. Survivors of Chinese ethnicity were more likely dissatisfied with cosmetic appearance compared to Malay survivors (22% and 14% respectively, adjusted OR = 2.4, 95%CI: 1.4–3.9). Women with DCIS (adjusted OR = 2.2, 95%CI: 1.3–3.7) or advanced stage disease (adjusted OR = 2.2, 95%CI: 1.2–3.9) had a higher risk of being dissatisfied with their cosmetic appearance. Women treated with mastectomy were at a higher risk of dissatisfaction with cosmetic appearance (adjusted OR = 1.7, 95%CI: 1.1–2.5). Dissatisfaction with cosmetic appearance was associated with increased depression scores. In this South-East Asian population, one in five breast cancer patients were (very) dissatisfied with the cosmetic outcome of treatment. Chinese women, women with larger tumors and women treated with mastectomy were most likely to report dissatisfaction with cosmetic appearance.

## Introduction

Breast cancer accounts for 18% of all cancers diagnosed in the Asia-Pacific region, where 42% of breast cancers are diagnosed in women under the age of 50 years [1]. Due to increasing incidence, and improving survival, the number of women living with consequenses of breast cancer and its treatment is increasing.

One of the main goals of breast cancer surgery is to achieve high cure rates, with satisfying cosmetic results. Over the past decades, surgery has become increasingly less invasive with the introduction of breast-conserving surgery. Good cosmetic outcomes are important, as it has been shown that patient-reported satisfaction with cosmetic appearance is associated with better quality of life, lower depression, less stress, and lesser social and sexual avoidance behavior [2–6].

Research on cosmetic outcome after breast cancer treatment is mainly done in Western populations, and has hardly been studied in Asia. However, the more advanced stages at diagnosis in Asian women, the smaller breast size, different health beliefs and behavior might result in differences in satisfaction with cosmetic appearance [7, 8].

In this study, we studied prevalence of, and demographic and clinical factors associated with the level of satisfaction with cosmetic appearance after surgery for breast cancer in the multi-ethnic Asian population of Singapore. In addition, we investigated the association between dissatisfaction with cosmetic appearance and patient-reported anxiety, depression, global health status, and pain.

## Materials and methods

This cross-sectional study was conducted at the National University Hospital (NUH), Singapore, between April 2014 and April 2015 and received institutional ethical approval. Female breast cancer survivors, who were at least one year post diagnosis and visiting outpatient clinics for routine follow-up, were eligible for inclusion in the current study. Participants were selected from the daily patient list of the Breast Care Centre, the Breast Imaging Centre, and the Oncology Centre. Breast cancer survivors who were unable to communicate in English or Mandarin or who had psychological disorders other than anxiety and depression recorded in their medical records were excluded.

We invited 631 breast cancer survivors to participate in the study of which 409 (65%) agreed. Of the 222 women that refused participation or did not return the questionnaire, 107 (48%) were not interested in the research topic, 37 (17%) did not have time to participate, 21 (9%) indicated that they were not interested to participate as they did not experience any adverse effects, 12 (5%) agreed to participate but did not return the questionnaire, 12 (5%) did not participate because their accompanying relatives disagreed, 12 (5%) felt that the questions were too personal, 11 (5%) indicated to be too tired, 6 (3%) had hearing or speaking difficulties which made comprehension of questionnaire difficult, and 5 (2%) commented that the questionnaire was too long. For this study, an additional 25 participants were excluded– 11 survivors of other ethnicities (including Caucasians and women of European descent), 10 who did not complete the cosmetic evaluation questionnaire, and 4 who did not have surgery.

The validated cosmetic evaluation questionnaire measures patients’ satisfaction with overall cosmetic appearance and satisfaction with cosmetic appearance of the treated breast compared to the untreated breast [9]. Here, satisfaction with cosmetic appearance was measured using a single item ‘How satisfied/dissatisfied are you with the appearance of the treated breast compared with the untreated breast?’ of the questionnaire [9]. Participants with bilateral surgery were asked to compare the treated breasts with their pre-treatment appearances.

In addition to the cosmetic evaluation questionnaire, the Hospital Anxiety and Depression Scale (HADS), European Organisation for Research and Treatment of Cancer–Quality of Life Questionnaire–Core 30 (EORTC-QLQ-C30, version 3), and the corresponding breast cancer module (EORTC-QLQ-BR23, version 1) were administered [10–13]. The scores for each questionnaire were calculated as recommended by their respective scoring manuals. Satisfaction with cosmetic appearance was correlated with concerns about body image, as measured by the EORTC-QLQ-BR23 (Spearman’s correlation coefficient -0.269, p-value < 0.001).

At baseline, we collected the following information from the NUH Breast Cancer Registry for 296 (77%) breast cancer patients diagnosed between 1990 and 2011 [7], and from the medical records for 88 (23%) breast cancer patients diagnosed after 2011: age at breast cancer diagnosis, ethnicity (Chinese, Malay, Indian), recurrent disease (i.e. occurring prior to recruitment and after completion of treatment; classified as yes or no), tumor size (mm), stage at diagnosis (*in situ*, stage I–IV, unknown), tumor differentiation grade (poor, moderate, good, unknown), estrogen receptor status (positive >1% of tumour cells expressing estrogen receptors, negative, unknown), type of surgery based on the most recent surgery (breast conserving surgery, mastectomy, mastectomy with reconstruction), axillary staging (axillary dissection, sentinel node biopsy only/none, based on the most recent surgery), radiotherapy (yes, no, unknown), (neo)adjuvant chemotherapy (yes, no, unknown), and hormone therapy (yes, no, unknown). Treatment details and tumor characteristics were based on treatment shortly after diagnosis, unless otherwise stated.

### Statistical analysis

The 5 categories of satisfaction with cosmetic appearance were re-categorized into 3 categories (1. very satisfied/ satisfied, 2. not dissatisfied, and 3. dissatisfied/ very dissatisfied) to univariably test the association between satisfaction with cosmetic appearance and demographic and clinical factors. This was done using the Kruskal-Wallis test for continuous variables and Chi-square test or Fisher’s exact test for categorical variables. Cumulative logit model maintains the order of the 5 categories of satisfaction with cosmetic outcome without restricting the interpretation of the difference between any two consecutive categories to be the same [14]. This multivariable model was used to analyze the association of ethnicity (Chinese, Malay, and Indian), tumor stage (*in situ*, stage I/II, and III/IV), and type of surgery (breast conserving surgery, mastectomy, mastectomy with reconstruction) with satisfaction with cosmetic appearance. Type of surgery while not statistically significant in the univariable analysis was included as studies have shown it to be a potential confounder. The Fisher’s exact test was used to analyze the association between the level of satisfaction with cosmetic appearance and anxiety and depression (HADS). The association between satisfaction with cosmetic appearance and global health status and pain (EORTC-QLQ-C30) was analyzed using Kruskal-Wallis test. Subgroup analyses, assessing the associations between satisfaction with cosmetic appearance and ethnicity and stage were performed in survivors who had (i) mastectomy only, and (ii) breast conserving surgery. Statistical analyzes were performed with R version 3.2.3. Missing data was between 0% and 15% (estrogen receptor status), available case analysis was used.

## Results

A total of 384 breast cancer survivors were included of which 292 (76%) were Chinese, 71 (19%) were Malay, and 21 (5%) were Indian. Chinese participants had the oldest median age at diagnosis (52 years), followed by Malays (49 years), and Indians (46 years) (Table 1). The median time since diagnosis was 5 years for Chinese survivors and 4 years for Malay and Indian survivors. Tumor stage at diagnosis varied by ethnicity, with 90%, 80%, and 67% of Chinese, Malay, and Indian survivors, respectively, diagnosed with early stage breast cancer (*in situ*, stage I or II). Otherwise survivors of different ethnicities were similar in terms of tumor characteristics and treatments (Table 1).

**Table 1:** Demographics, tumor characteristics, and treatments of 292 breast cancer patients of Chinese ethnicity, 72 of Malay ethnicity, and 21 of Indian ethnicity.

	Ethnicity			
	Chinese	Malay	Indian	
	292 (76%)	71 (18%)	21 (5%)	P-value
**Median age at diagnosis (IQR)**	52 (45–57)	49 (43–54)	46 (42–55)	0.016
**Median age at time of survey (IQR)**	59 (52–65)	53 (48–57)	49 (47–63)	<0.001
**Median time since diagnosis (IQR)**	5 (3–9)	4 (2–8)	4 (3–6)	0.004
**Recurrence**^**a**^				
Yes	24 (8)	6 (8)	0 (0)	0.589
No	268 (92)	65 (92)	21 (100)	
**Tumor stage**				
*In situ*	45 (15)	6 (8)	4 (19)	0.002
I/II	220 (75)	51 (72)	10 (48)	
III/IV	24 (8)	14 (20)	6 (29)	
*Unknown*	3 (1)	0 (0)	1 (5)	
**Tumor grade**				
Well differentiated	45 (15)	9 (13)	1 (5)	0.402
Moderately differentiated	114 (39)	30 (42)	7 (33)	
Poorly differentiated	118 (40)	29 (41)	13 (62)	
*Unknown*	15 (5)	3 (4)	0 (0)	
**Estrogen receptor status**				
Positive	186 (64)	50 (70)	12 (57)	0.796
Negative	70 (24)	17 (24)	6 (29)	
*Unknown*	36 (12)	4 (6)	3 (14)	
**Type of surgery**^**b**^				
Breast conserving surgery	120 (41)	22 (31)	9 (43)	0.574
Mastectomy	147 (50)	43 (61)	11 (52)	
Mastectomy with reconstruction	25 (9)	6 (8)	1 (5)	
**Axillary Clearance**^**b**^				
Axillary dissection	170 (58)	51 (72)	13 (62)	0.101
Sentinel node biopsy only/none	122 (42)	20 (28)	7 (33)	
*Unknown*	0 (0)	0 (0)	1 (5)	
**Radiotherapy**				
Yes	174 (60)	38 (54)	16 (76)	0.192
No	106 (36)	28 (39)	4 (19)	
*Unknown*	12 (4)	5 (7)	1 (5)	
**(Neo) adjuvant chemotherapy**				
Yes	136 (47)	30 (42)	11 (52)	0.669
No	146 (50)	38 (54)	9 (43)	
Unknown	10 (3)	3 (4)	1 (5)	
**Hormone therapy**				
Yes	196 (67)	55 (77)	13 (62)	0.099
No	88 (30)	12 (17)	6 (29)	
*Unknown*	8 (3)	4 (6)	2 (10)	

‘Unknown’ were excluded from statistical testing.

^a^ Fisher’s Exact test was used.

^b^ Not based on treatment at diagnosis but on final treatment received any time before the survey.

IQR: Interquartile range.

Overall, 77 out of 384 (20%) breast cancer survivors were (very) dissatisfied with their cosmetic appearance. Chinese breast cancer survivors had the highest proportion of women who were (very) dissatisfied with their cosmetic appearance (63/292, 22%), followed by Indian (4/21, 19%) and Malay (10/71, 14%) (Table 2).

**Table 2:** Demographics, tumor characteristics, and treatments of 384 breast cancer patients by satisfaction with cosmetic appearance.

	Satisfaction with Cosmetic Appearance			
	Very Satisfied / Satisfied	Not Dissatisfied	Dissatisfied / Very Dissatisfied	
	n = 199 (52%)	n = 108 (28%)	n = 77 (20%)	P-value
**Ethnicity**				
Chinese	134 (46)	95 (33)	63 (22)	0.001
Malay	51 (72)	10 (14)	10 (14)	
Indian	14 (67)	3 (14)	4 (19)	
**Age at diagnosis**^**a**^	50 (44–57)	50 (45–58)	51 (45–55)	0.717
**Age at time of survey**^**a**^	57 (49–64)	59 (52–64)	58 (52–63)	0.330
**Time since diagnosis**^**a**^	5 (3–9)	6 (3–9)	5 (3–9)	0.289
**Recurrence**^**b**^				
Yes	15 (50)	8 (27)	7 (23)	0.877
No	184 (52)	100 (28)	70 (20)	
**Tumor stage**				
*In situ*	19 (35)	23 (42)	13 (24)	0.021
I/II	159 (57)	70 (25)	52 (19)	
III/IV	19 (43)	13 (30)	12 (27)	
*Unknown*	2	2	0 (0)	
**Tumor grade**				
Well differentiated	28 (51)	14 (25)	13 (24)	0.780
Moderately differentiated	82 (54)	39 (26)	30 (20)	
Poorly differentiated	80 (50)	50 (31)	30 (19)	
*Unknown*	9 (50)	5 (28)	4 (22)	
**Estrogen receptor status**				
Positive	137 (55)	64 (26)	47 (19)	0.688
Negative	47 (51)	28 (30)	18 (19)	
*Unknown*	15 (35)	16 (37)	12 (28)	
**Type of surgery**^**c**^				
Breast conserving surgery	86 (57)	40 (26)	25 (17)	0.286
Mastectomy	95 (47)	62 (31)	44 (22)	
Mastectomy with reconstruction	18 (56)	6 (19)	8 (25)	
**Axillary Clearance**^**c**^				
Axillary dissection	121 (52)	66 (28)	47 (20)	1.000
Sentinel node biopsy only/none	77 (52)	42 (28)	30 (20)	
*Unknown*	1 (100)	0 (0)	0 (0)	
**Radiotherapy**				
Yes	118 (52)	66 (29)	44 (19)	0.874
No	72 (52)	37 (27)	29 (21)	
*Unknown*	9 (50)	5 (28)	4 (22)	
**(Neo) adjuvant chemotherapy**				
Yes	94 (53)	45 (25)	38 (21)	0.460
No	97 (50)	60 (31)	36 (19)	
Unknown	8 (57)	3 (21)	3 (21)	
**Hormone therapy**				
Yes	141 (53)	72 (27)	51 (19)	0.642
No	51 (48)	33 (31)	22 (21)	
*Unknown*	7 (50)	3 (21)	4 (29)	

^a^ Median (interquartile range).

‘Unknown’ were excluded from statistical testing.

^b^ Fisher’s Exact test was used.

^c^ Not based on treatment at diagnosis but on final treatment received any time before the survey.

Satisfaction with cosmetic appearance was associated with tumor stage: among survivors diagnosed with stage I/II breast cancer 19% were (very) dissatisfied, while 27% of survivors diagnosed with stage III/IV breast cancer were (very) dissatisfied (Table 2). Of the survivors diagnosed with *in situ* breast cancer 24% were (very) dissatisfied (Table 2).

After adjustment for tumor stage and type of surgery, ethnicity remained independently associated with satisfaction with cosmetic appearance (Table 3). Survivors of Malay and Indian ethnicities were 0.4 (95% CI: 0.3–0.7 and 0.2–1.0, respectively) times as likely to report being (very) dissatisfied with cosmetic appearance compared with Chinese survivors. Survivors diagnosed with *in situ* and survivors with stage III/IV breast cancer were 2.2 (95% CI: 1.3–3.7 and 1.2–1.6, respectively) times as likely to report being (very) dissatisfied with cosmetic appearance compared to survivors who were diagnosed with stage I/II breast cancer. Compared to survivors who had breast conserving surgery, survivors treated with mastectomy were 1.7 (95% CI: 1.1–2.5) times as likely to report (very) dissatisfied with cosmetic appearance. Mastectomy with reconstruction was not significantly associated with satisfaction with cosmetic appearance (OR: 1.3, 95% CI: 0.6–2.7).

**Table 3:** Association of ethnicity with satisfaction with cosmetic appearance using cumulative logit models, adjusted for tumor stage and type of surgery in (i) 380 breast cancer patients, (ii) subgroup of 199 patients who had mastectomy only, and (iii) subgroup of 139 patients who were diagnosed with *in situ*, stage I/II breast cancer and had breast conserving surgery. Satisfaction with cosmetic appearance is on a scale of 1 to 5, where 1 is satisfied and 5 is very dissatisfied.

	(i) All^a^, n = 380				(ii) TM, n = 199		(ii) BCS^b^, n = 139	
	Univariable		Multivariable		Multivariable		Multivariable	
Determinants	OR (95% CI)	P-value	OR (95% CI)	P-value	OR (95% CI)	P-value	OR (95% CI)	P-value
Ethnicity								
Chinese	Ref	–	Ref	–	Ref	–	Ref	–
Malay	0.5 (0.3–0.7)	0.001	0.4 (0.3–0.7)	<0.001	0.4 (0.2–0.8)	0.012	0.6 (0.3–1.6)	0.322
Indian	0.5 (0.2–1.2)	0.121	0.4 (0.2–1.0)	0.050^d^	0.7 (0.2–2.2)	0.526	0.5 (0.1–1.9)	0.292
Tumor stage								
*In situ*	2.1 (1.3–3.5)	0.004	2.2 (1.3–3.7)	0.003	4.0 (1.8–9.1)	<0.001	1.6 (0.8–3.3)	0.214
I/II	Ref	–	Ref	–	Ref	–	Ref	–
III/IV	2.0 (1.1–3.5)	0.020	2.2 (1.2–3.9)	0.008	2.9 (1.4–6.1)	0.004	–	–
Type of surgery^c^								
Breast conserving surgery	Ref	–	Ref	–	–	–	–	–
Mastectomy	1.6 (1.1–2.3)	0.024	1.7 (1.1–2.5)	0.009	–	–	–	–
Mastectomy with reconstruction	0.8 (0.4–1.7)	0.584	0.8 (0.4–1.6)	0.459	–	–	–	–

^a^ 4 patients with unknown stage were excluded.

^b^ Patients who were diagnosed with *in situ*, *s*tage I/II breast cancer.

^c^ Based on last treatment received.

^d^ p-value = 0.0503.

BCS: Breast conserving surgery, TM: Mastectomy only, OR: Odds ratio, CI: Confidence interval, Ref: Reference level.

Patient-reported depression was associated with satisfaction with cosmetic appearance. The proportion of survivors with borderline abnormal and abnormal depression scores (score of 8–21) [13] was 16% and 8% respectively in those who were (very) satisfied, 19% and 5% respectively in those who were not dissatisfied, and 22% and 14% respectively in those who were (very) dissatisfied with cosmetic appearance, respectively (p<0.001). Patient-reported anxiety, global health status, and pain were not associated with satisfaction with cosmetic appearance (Table 4).

**Table 4:** Anxiety, depression, global health status, and pain of 384 breast cancer patients in relation to satisfaction with cosmetic appearance, in April 2014 to April 2015.

	Satisfaction with Cosmetic Appearance			
	Very Satisfied / Satisfied	Not Dissatisfied	Dissatisfied / Very Dissatisfied	
	199 (52%)	108 (28%)	77 (20%)	P-value
**Anxiety (HADS)**^**a**^				
0–7	151 (54)	82 (29)	49 (17)	0.120
8–10	32 (46)	20 (29)	17 (25)	
11–21	16 (50)	5 (16)	11 (34)	
**Depression (HADS)**^**a**^				
0–7	184 (57)	86 (27)	54 (17)	<0.001
8–10	10 (24)	15 (37)	16 (39)	
11–21	5 (28)	6 (33)	7 (39)	
**Global health status (EORTC-QLQ-C30)**^**b**^	75 (58–92)	71 (67–83)	67 (50–83)	0.052
**Pain (EORTC-QLQ-C30)**^**b**^	17 (0–17)	0 (0–17)	17 (0–33)	0.307

^**a**^ Score of 0–7 indicates normal level of anxiety/depression, score of 8–10 indicates borderline abnormal level, and 11–21 indicates abnormal level of anxiety/depression.

^**b**^ Median (interquartile range).

HADS: Hospital Anxiety and Depression Scale, EORTC-QLQ-C30: European Organisation for Research and Treatment of Cancer–Quality of Life Questionnaire–Core 30.

In our subgroup analysis of survivors who had mastectomy only (n = 199) and breast conserving surgery (n = 139), survivors diagnosed with *in situ* breast cancer were 4.0 (95% CI: 1.8–9.1) and 1.6 (95% CI: 0.8–3.3) times, respectively, more likely to be dissatisfied with cosmetic appearance as compared with survivors diagnosed with stage I/II breast cancer (Table 3).

## Discussion

In this South-East Asian population of breast cancer patients, one in five patients was (very) dissatisfied with cosmetic outcome after breast cancer treatment. Breast cancer survivors of Malay ethnicity were most likely to be satisfied, and Chinese survivors were the least satisfied. Accounting for ethnicity differences, being diagnosed with *in situ* or stage III/IV tumors, and having had mastectomy were independent risk factors of being dissatisfied with cosmetic appearance. Dissatisfaction with cosmetic appearance was associated with higher levels of depression.

Several other studies in Asia have described differences in body image, including the use of the Body Image Scale (BIS) and Body Image subscale of the EORTC-QLQ-BR23, however they did not focus on cosmetic satisfaction. Body image is a much broader domain including impact on general appearance, quality of life and femininity [15]. In our data, satisfaction with cosmetic appearance was correlated with the Body Image subscale of the EORTC-QLQ-BR23. The aim of our study was to assess dissatisfaction with cosmetic appearance after surgery for breast cancer in a multi-ethnic population. Hence we focused on satisfaction with cosmetic appearance alone, instead of the more complex body image.

Jagsi et al. did not observe differences in satisfaction with cosmetic outcomes between Whites, Blacks, and Hispanic breast cancer survivors in the United States [16]. However, we did find differences between women in multi-ethnic Asian population. Breast cancer survivors of Malay ethnicity were less likely to report dissatisfaction with cosmetic results as compared to Chinese survivors. On average, women of Malay ethnicity have larger breasts as compared to Chinese women [17], which may explain why they were less likely to be dissatisfied with cosmetic results.

Satisfaction with cosmetic appearance was worse both in patients with *in situ* and in patients with advanced stage cancer, probably due to the larger tumor volumes that needed to be removed in these stages as compared to stage I/II. In contrast, Falk et al. did not find differences in body image between survivors diagnosed with stage II /III breast cancer [18]. This may be due to a similar distribution of tumor size within the two groups (stage II vs III). Pickler et al. also did not find an association of body image with tumor stage (*in situ*/I/II vs. III/IV) [19]. The latter could be explained by the fact that including potentially larger *in situ* tumors in the same category as smaller stage I/II tumors increases the average tumor size and the average excised lump size in this category.

Having mastectomy only was associated with greater dissatisfaction with cosmetic appearance as compared with having breast conserving surgery. This is consistent with most previous studies in Western populations, where survivors with breast conserving surgery expressed greater satisfaction with general cosmetic outcome [16, 20, 21], better body image [18, 22–24], and less impact on body image [25, 26].

In survivors who had mastectomy, being diagnosed with *in situ* cancer was associated with a much increased risk of being dissatisfied with cosmetic appearance as compared with being diagnosed with stage III/IV cancer. Patients diagnosed with advanced stages are mainly focused on survival, while patients diagnosed with *in situ* cancer may focus more on side effects of treatment as they are aware of their good prognosis. The effect observed was also higher than that in all survivors. In addition, among survivors who had breast conserving surgery, stage was not significantly associated with satisfaction with cosmetic appearance.

Age and time since diagnosis were not associated with satisfaction with cosmetic appearance in our study. In concordance, Bober et al. did not find age at time of survey to be associated with appearance in survivors diagnosed with *in situ* breast cancer [27]. Falk et al. did not find an association between age at diagnosis or at time of survey with body image [18]. However others found that younger age at diagnosis and younger age at time of survey was associated with poorer body image [23, 24].

Dissatisfaction with cosmetic appearance was associated with increased depression scores, but not with anxiety, global health status or pain. Studies have found that survivors who had poorer body image (measured by BIS) [18], more body image concerns [2, 3, 25], or were less satisfied with their physical appearance [21] were more likely to have higher levels of depression.

Contrary to our results, Falk et al. found that poorer body image was associated with higher levels of anxiety [18]. However, most other studies also did not find an association between satisfaction with cosmetic appearance or body image and anxiety [21, 24, 25]. McClelland et al. and Begovic-Juhant et al. found that more concerns about body image were associated with poorer global health status [2, 5]. However the direction of the relation between satisfaction with cosmetic outcomes and mental health or global health status cannot be determined in these cross-sectional studies [2, 3, 5, 24]. Without knowing the temporal occurrence, it cannot be determined if dissatisfaction with cosmetic appearance causes depression or poorer global health status, or that women who are depressed or have poorer global health status are more likely to be dissatisfied with cosmetic appearance.

We acknowledge that our study suffers from several limitations. It is a single centre study in Singapore which may have repercussions on its generalizability. The majority of our survivors are of Chinese ethnicity, however Singapore is a multiethnic city, the proportion of survivors of non-Chinese ethnicity is substantial. Due to the cross-sectional design it is unclear whether higher levels of depression results in survivors being dissatisfied with appearance, or whether being dissatisfied with appearance leads to higher levels of depression. Survivors were more satisfied with cosmetic appearance than patients shortly after treatment. The value that survivors place on cosmetic appearance and obtaining the cosmetic results that matches the survivors’ expectation has been shown to be associated with satisfaction [6, 21, 28, 29]. Enabling patients to have realistic expectation of post treatment appearance and engaging the patient in shared decision making could reduce the risk of dissatisfaction.

## Conclusions

Twenty percent of breast cancer survivors in Singapore report dissatisfaction with cosmetic appearance following breast cancer surgery, and dissatisfaction with cosmetic outcome is most common among Chinese women. Being diagnosed with *in situ* or stage III/IV breast cancer and having had mastectomy as compared with breast conserving surgery, were independent determinants of dissatisfaction with cosmetic appearance. Prospective studies are needed to assess if interventions aimed at improving information of patients and expectation management, such as shared decision making and joining support groups, may improve satisfaction with appearance and reduce depression.
